# NGS_SNPAnalyzer: a desktop software supporting genome projects by identifying and visualizing sequence variations from next-generation sequencing data

**DOI:** 10.1007/s13258-020-00997-7

**Published:** 2020-09-26

**Authors:** Dong-Jun Lee, Taesoo Kwon, Chang-Kug Kim, Young-Joo Seol, Dong-Suk Park, Tae-Ho Lee, Byung-Ohg Ahn

**Affiliations:** 1 Genomics Division, National Institute of Agricultural Science, 370 Nongsaengmyeong-ro, Jeonju, 54874 Republic of Korea; 2 Interdisciplinary Program in Bioinformatics, Seoul National University, 1 Gwanak-ro, Gwanak-gu, Seoul, 08826 Republic of Korea; 3 Gene Engineering Division, National Institute of Agricultural Science, 370 Nongsaengmyeong-ro, Jeonju, 54874 Republic of Korea

**Keywords:** Next-generation sequencing, Whole-genome sequencing, Variant identification, Genomics, Pipeline

## Abstract

**Background:**

Sequence variations such as single nucleotide polymorphisms are markers for genetic diseases and breeding. Therefore, identifying sequence variations is one of the main objectives of several genome projects. Although most genome project consortiums provide standard operation procedures for sequence variation detection methods, there may be differences in the results because of human selection or error.

**Objective:**

To standardize the procedure for sequence variation detection and help researchers who are not formally trained in bioinformatics, we developed the NGS_SNPAnalyzer**,** a desktop software and fully automated graphical pipeline.

**Methods:**

The NGS_SNPAnalyzer is implemented using JavaFX (version 1.8); therefore, it is not limited to any operating system (OS). The tools employed in the NGS_SNPAnalyzer were compiled on Microsoft Windows (version 7, 10) and Ubuntu Linux (version 16.04, 17.0.4).

**Results:**

The NGS_SNPAnalyzer not only includes the functionalities for variant calling and annotation but also provides quality control, mapping, and filtering details to support all procedures from next-generation sequencing (NGS) data to variant visualization. It can be executed using pre-set pipelines and options and customized via user-specified options. Additionally, the NGS_SNPAnalyzer provides a user-friendly graphical interface and can be installed on any OS that supports JAVA.

**Conclusions:**

Although there are several pipelines and visualization tools available for NGS data analysis, we developed the NGS_SNPAnalyzer to provide the user with an easy-to-use interface. The benchmark test results indicate that the NGS_SNPAnayzer achieves better performance than other open source tools.

**Electronic supplementary material:**

The online version of this article (10.1007/s13258-020-00997-7) contains supplementary material, which is available to authorized users.

## Introduction

Massive parallel sequencing has been successful in identifying causal genes of some diseases by detecting sequence variation. Because of this, next-generation sequencing (NGS) is popular in all aspects of life sciences. For example, in Mendelian diseases such as the Freeman–Sheldon syndrome (Ng et al. 2009), Miller syndrome (Ng et al. 2010b), and some complex diseases such as the Kabuki syndrome (Ng et al. 2010a), the introduction of NGS technology resulted in the successful detection of causal variants of the diseases. In agricultural science, crop (Yu et al. 2011) and cattle (Schaeffer 2006) breeding using NGS-produced molecular markers have been trialled. An ultra-high-density genetic map was constructed, which significantly reduced the breeding cost. Based on the success of NGS in genome research, the identification of sequence variations, such as single nucleotide variants and small insertions and deletions (INDELs), became one of the main objectives of genome projects. To support the detection of sequence variations, the variant detection procedures are implemented as a standard operation procedure (SOP), and the corresponding consortium provides a shell script (https://github.com/ekg/1000G-integration). On the other hand, several tools that analyse NGS data have been developed; such analysis includes quality control (QC), mapping, variation calling, variation annotation, and format conversion. However, the lack of tool integration and the many options included in their functionality often confuses the user when considering the input and output of the tools and their compatibility. To overcome this inconvenience, several pipelines and workflows have been developed by the commercial and open-source communities. NGS pipelines such as ngs_backbone (Blanca et al. 2011) and GATK (McKenna et al. 2010) provide simple commands to perform a complete NGS data analysis. Depending on the user’s purpose, GATK provides a more detailed command in every step of the analysis. As a workflow, Galaxy (Goecks et al. 2010) and CLC genomics workbench provide the user with easy-to-use graphical user interfaces (GUIs). In spite of the rush in the development of pipelines and integrated environments, each has their own strengths and limitations (Table 1).

**Table 1:** Comparison of SNP analysis pipelines in terms of user-friendly graphic interface and OS

Function	Name						
	Category	Annovar	Ngs_backbone	inGAP	Galaxy	CLC genomics workbench	NGS_SNPAnalyzer
Analysis	Quality control (QC)	–	Ο	Ο	Ο	Ο	Ο
	Read mapping	–	Ο	Ο	Ο	Ο	Ο
	Variant call	–	Ο	Ο	Ο	Ο	Ο
	Variant annotation	Ο	–		Ο	Ο	Ο
	Visualzation	–	–	–	–	–	Ο
	Manual mode	Ο	Ο	Ο	–	Ο	Ο
	Batch mode	–	–	–	Ο	Ο	Ο
User interface	Graphic user interface	–	–	Ο	Ο	Ο	Ο
	Unix/Linux	Ο	Ο	Ο	Ο	Ο	Ο
	Window	–	–	–	–	Ο	Ο
	Mac	–	–	–	–	Ο	–
	HPC support	–	Ο	–	Ο	–	–

*HPC* high performance computing

Most pipelines offer only a command–line interface in which the user needs to be familiar with Unix/Linux commands. Moreover, the user must obtain a file transport protocol (ftp) connection to upload the data files and secure shell (ssh) capabilities for secure terminal login, even while using their own personal computers to analyse the NGS data. In addition, the integrated environments do not support batch processes for mass production of the genotype. To support the SOP for sequence variation detection and provide the user with a convenient graphical environment, we developed a desktop software, the NGS_SNPAnalyzer. NGS_SNPAnalyzer includes all the functionalities for variant detection: QC, mapping, filtering, variant calling, and visualization. It has two modes of action: a batch job mode to support batch identification of variants, and a step-by-step mode to verify the result of each step. It can be executed using pre-set pipelines and options; however, it can also be customized via user-specified options. In addition, the NGS_SNPAnalyzer can be installed on any operating system (OS) that supports JAVA, such as Windows, Linux, and MacOS.

## Methods

### Tools used in the pipeline

**Fig. 1:**
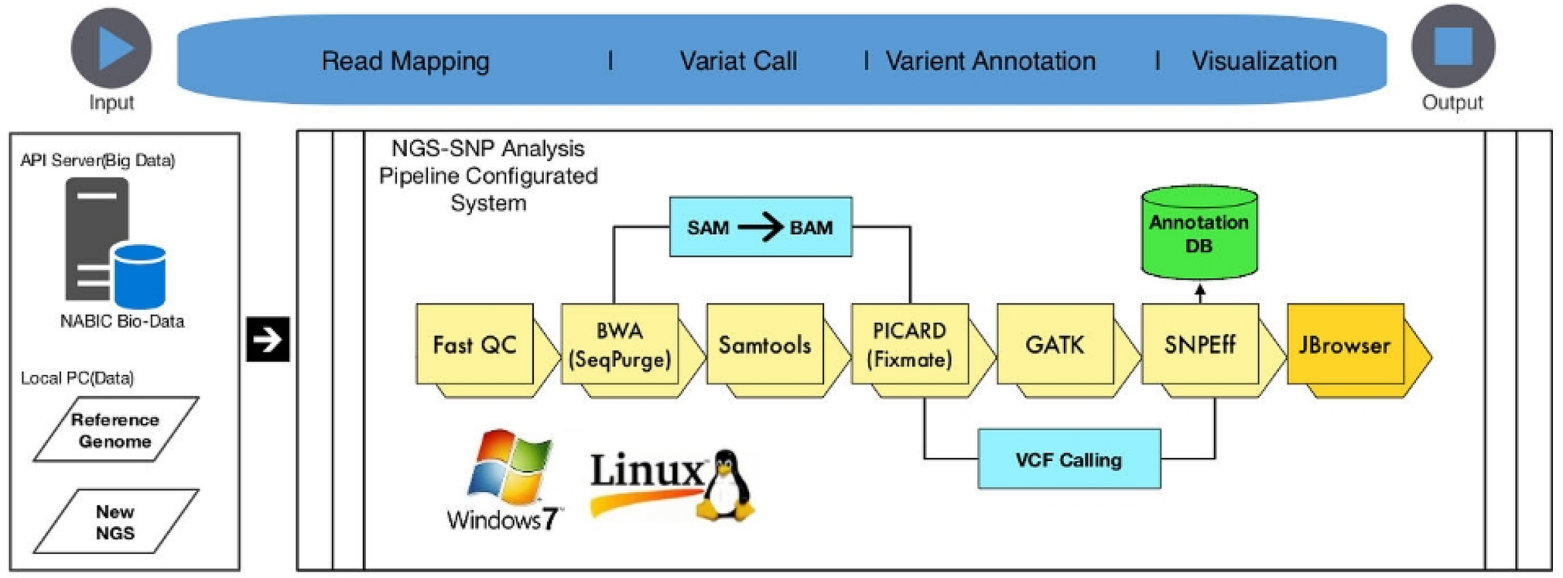
NGS data analysis pipeline used in the NGS_SNPAnalyzer

NGS_SNPAnalyzer includes FastQC (version 0.11.5) for QC. For quality filtering and trimming of sequence reads, the NGS_SNPAnalyzer employs TrimmOmatic (version 0.36) (Bolger et al. 2014). For read mapping, BWA (version 0.7.16a) (Li and Durbin 2009) is used. SAMtools (Li 2011) is used for file format conversion and indexing. For fixing mate-pair information and removing duplicate reads, Picard (version 2.9.4) is used. The SNP/INDEL identification and annotation tools used in the NGS_SNPAnalyzer are the Genome Analysis Toolkit (version 3.7.0) (McKenna et al. 2010) and SnpEff (version 4.3q) (Cingolani et al. 2012), respectively. JBrowser (version 1.12.3) (Skinner et al. 2009) is used for the visualization of identified variants. All the tools integrated in the NGS_SNPAnalyzer are summarized in Table 2. The SOP for sequence variation detection, which is included in NGS_SNPAnalyzer, was developed according to the pipeline of the National Agricultural Biotechnology Information Center (NABIC, Republic of Korea) (Fig. 1).

**Table 2:** Tools included in NGS_SNPAnalyzer

Step	Tool	Version	Reference
QC	FastQC	0.11.5	(https://www.bioinformatics.babraham.ac.uk/projects/fastqc/)
	TrimmOmatic	0.36	Bolger et al. (2014)
Alignment	BWA	0.7.16a	Li and Durbin (2009)
Post-processing	Samtools	0.1.18	Li (2011)
	Picard	2.9.4	(https://broadinstitute.github.io/picard/)
	BamTools	2.4.2	(https://github.com/pezmaster31/bamtools)
	GATK(IndelRealigner)	3.7.0	McKenna et al. (2010)
Variant call	GATK(HaplotypeCaller)	3.7.0	McKenna et al. (2010)
	GATK(UnifiedGenotyper)	3.7.0	McKenna et al. (2010)
Variant annotation	SnpEff	4.3q	Cingolani et al. (2012)
Visualization	Jbrowser	1.12.3	Skinner et al. (2009)

*QC* quality control

### Implementation

NGS_SNPAnalyzer is implemented using JavaFX (version 1.8) so that it is not limited for use on any specific OS. However, the tools employed in the NGS_SNPAnalyzer were compiled on Microsoft Windows (version 7, 10) and Ubuntu Linux (version 16.04, 17.0.4). For Microsoft Windows, the Cygwin (version 2.88.1) library was used to compile the tools included in NGS_SNPAnalyzer. The GNU compiler collection version 6.3.0 for Microsoft Windows and version 7.2.0 for Ubuntu Linux were used as C-language compilers.

## Results and discussion

Users can access all NGS_SNPAnalyzer functions using two modes: step-by-step and one-step.

### Create project and import input files

Before selecting the mode, the user must create a project and specify the data files: fastq files of sequencing reads and a reference file in FASTA format (Suppl. 2a). Currently, NGS_SNPAnalyzer only accepts fastq files produced by the Illumina platform. To move to the next step, the user must specify a folder location where the project file would be saved and provide a project name. To support genome projects, NGS_SNPAnalyzer can download a reference file from the corresponding genome project server, NABIC, through the application programme interface provided by the genome project. When the user selects a reference file, NGS_SNPAnalyzer investigates the index file of the reference sequence. If the reference file is not indexed, NGS_SNPAnalyzer will perform the indexing of the reference file.

### Step-by-step mode

Using the step-by-step mode, the user can check every step of the NGS data analysis process and change or execute each option during each step (Suppl. 2b). The NGS_SNPAnalyzer provides the user with a log window to monitor the progress of the step. If the user changes any option in the step, the selected option will be the default option during the same step in each subsequent run.

### One-step mode

The step-by-step mode is an easy way to perform and observe the NGS data analysis results using the NGS_SNPAnalyzer. However, the user is required to run each step manually and wait until the step ends, slowing down the NGS data analysis and causing an inconvenience. Therefore, the one-step mode can run all the processes employed in NGS_SNPAnalyzer using a single click. The one-step mode only stops at the end of the NGS data analysis (Suppl. 2c), which is the visualization step using JBrowser. The user can monitor the results of the NGS data analysis via the log window. Moreover, the user can customize the detailed options used in the one-step mode as desired.

### Quality control

Quality control and filtering are necessary in genomic variation detection from the NGS data because of their higher sequencing error rates when compared to the Sanger method (Nowrousian 2010). NGS_SNPAnalyzer uses FastQC (version 0.11.5) to check the quality of the sequence reads before and after QC. For quality control of the sequence reads, TrimmOmatic (version 0.36) (Bolger et al. 2014) is employed. The sequence reads under the score [Phred (Ewing and Green 1998)] specified by the user will be filtered out and low-quality regions in 5′- and 3′-ends can be trimmed using TrimmOmatic. The user can also specify the regions that should be trimmed.

### Read mapping and duplicate removal

BWA (version 0.7.16a) (Li and Durbin 2009) is used for short read mapping to the reference sequence. After the short read mapping, the resulting file will be converted from sequence alignment map (sam) to binary alignment map (bam) format, then sorted and indexed by SAMtools (Li 2011). To verify and fix mate-pair information, the Fixmate command of Picard (version 2.9.4) is used. Duplicate reads are removed using the MarkDuplicates and AddOrReplaceReadGroups commands of Picard. Before and after fix mate and removal of duplicate reads, the statistics of sequence reads is reported by BamTools (Barnett et al. 2011) in the log window.

### SNP/INDEL identification

The Genome Analysis Toolkit (version 3.7.0) (McKenna et al. 2010) is used for single nucleotide polymorphism/INDEL (SNP/INDEL) identification in the NGS_SNPAnalyzer. It is mandatory for the UnifiedGenotyper that performs IndelRealigner to realign reads around the INDELs. Therefore, NGS_SNPAnalyzer uses the RealignerTargetCreator and IndelRealigner commands of GATK for this step. UnifiedGenotyper is a variant caller of NGS_SNPAnalyzer that identifies the SNPs and INDELs from the realigned reads.

### Variant annotation

The identified variants are annotated using SnpEff (version 4.3q) (Cingolani et al. 2012), and the functional effects of the variants on the genes are predicted. For *Arabidopsis thaliana* genome analysis, for example, NGS_SNPAnalyzer only includes the *Arabidopsis thaliana* database [TAIR10 genome (Swarbreck et al. 2008)]. For other organisms and non-model organisms, the SnpEff database should be included for the appropriate organism if it is available or the database should be generated using a genome annotation file in gff3 format and the reference sequence. After the variant annotation, the annotation statistics will be reported in the next step.

### Variant visualization

**Fig. 2:**
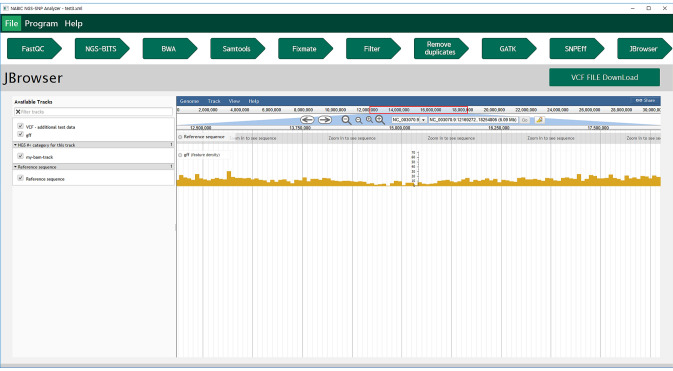
Visualization of variants: a total of four feature tracks are listed on the left panel of the genome browser: reference sequence, annotation information of reference in GFF format, mapped reads, and annotated variants

NGS_SNPAnalyzer displays the identified and annotated variants using JBrowser (version 1.12.3) (Skinner et al. 2009) (Fig. 2). A total of four feature tracks: reference sequence, annotation information of reference in GFF format, mapped reads, and annotated variants, are provided in the genome browser. The user can select what they want to display by clicking the check box of the corresponding feature tracks. The reference sequence and annotation information should be customized for the individual genome project or organism because it is only available for *Arabidopsis thaliana* in the current version of NGS_SNPAnalyzer. Meanwhile, the user can download the annotated variant profile by clicking the VCF file download button on the top-right of the genome browser to use for further analysis.

### Software benchmarking

To benchmark the software, we downloaded the complete *Arabidopsis thaliana* genome sequencing data under the accession number SRR519473 from the DNA Data Bank of Japan (DDBJ) FTP site. The data were generated by the *Arabidopsis thaliana* 1001 genomes project (https://1001genomes.org) (Long et al. 2013) using the Illumina HiSeq 2000 platform. The paired-end run includes 52,154,720 reads, and the number of bases is 10,430,944,000 bp. The dataset was mapped to the whole genome sequence of *Arabidopsis thaliana* (Accession: GCF_000001735.4, TAIR version 10) that was downloaded from The Arabidopsis Information Resource (TAIR) FTP site. To show the general usability of the software, we performed another benchmark on human exome sequencing data. We retrieved the exome sequencing data of NA19098 (Abecasis et al. 2010) under the accession number SRR077453 (22,555,779 reads) from the 1,000 genomes project consortium ftp site (ftp://ftp.1000genomes.ebi.ac.uk/vol1/ftp/).

We compared the NGS_SNPAnalyzer with the ngs_backbone pipeline on a Xeon server. Detailed specifications of the Xeon server and the test results are listed in Table 3. The NGS_SNPAnalyzer took 20 h 29 m 23 s from raw reads to variant annotation or visualization, whereas ngs_backbone took 49 h 51 min 12 s on the complete *Arabidopsis thaliana* genome sequencing data.

**Table 3:** Comparison of performance of pipelines

Pipelines	CPUs	RAM	Storage	OS	Times(hh:mm:ss)	
		(Gbytes)	(Gbytes)		*Arabidopsis thaliana*	*Homo sapiens*
NGS_SNPAnalyzer	12	32	220	Ubuntu 16.04.5	20:29:23	34:29:23
ngs_backbone*					49:51:12	82:51:48

*Because ngs_backbone has no visualization functionality, visualization process was not tested in the ngs_backbone pipeline

## Conclusion

Thus far, there are several pipelines and visualization tools for NGS data analysis and genome projects. However, most of them are general-purpose and are not customizable for a specific organism. They are not user-friendly and do not integrate all the tools required for genome analysis. The NGS_SNPAnalyzer is a user-friendly software for researchers who are not familiar with the command line interface used in SNP identification from NGS data. Additionally, the NGS_SNPAnalyzer is not OS-dependent because it is implemented using JavaFX. Unlike most open source software for NGS data analysis, the NGS_SNPAnalyzer provides the user with an easy-to-use interface and helps detect variations from the NGS data and explore variants genome-wide. The benchmark test on the complete *Arabidopsis thaliana* genome sequencing data demonstrated that the overall time consumed by the NGS_SNPAnalyzer was 2.43 times faster than ngs_backbone. In summary, the NGS_SNPAnalyzer shows better performance than other open source tools and provides researchers with an easy-to-use GUI to analyse NGS data.

### Outlook

Currently, the NGS_SNPAnalyzer does not provide the user with a multi-sample NGS data analysis. The functionality to allow multi-sample NGS data analysis will be included in the next version of the software.

## Electronic supplementary material

Below is the link to the electronic supplementary material.

Supplementary file1 (DOCX 16 kb)

Supplementary file2 (PPTX 1871 kb)
