# Supplementary material for: NGS_SNPAnalyzer: a desktop software supporting genome projects by identifying and visualizing sequence variations from next-generation sequencing data
Source: Genes Genomics. 2020 Sep 26;42(11):1311–7. doi: 10.1007/s13258-020-00997-7 (PMC7567733; doi:10.1007/s13258-020-00997-7)
Supplement: Supplementary file 1 — Supplementary file1 (DOCX 16 kb) [file 13258_2020_997_MOESM1_ESM.docx]

# Availability and requirements

# Project name: NGS_SNPAnalyzer

# Project home page: https://sourceforge.net/projects/ngs-snpanalyzer/

# Operating system(s): Windows or Linux

# Programming language: JavaFX

# Other requirements: All Perl libraries are listed in Supplementary.

# License: GNU General Public License

# Any restrictions to use by non-academics: license needed.

It is needed to install for running NGS_SNPAnalyzer.

1. Recent version of Strawberry Perl
2. Heap::Simple::XS module (prepare-refseqs.pl)

perl -MCPAN -e ‘force install Heap::Simple::XS‘

or cpan –f Heap::Simple::XS (Please, ignore some errors)

1. Devel::Size module and PerlIO::gzip module (flatfile-to-json.pl)

perl -MCPAN -e 'install Devel::Size‘

perl -MCPAN -e 'install PerlIO::gzip'
