# Supplementary material for: NGS_SNPAnalyzer: a desktop software supporting genome projects by identifying and visualizing sequence variations from next-generation sequencing data
Source: Genes Genomics. 2020 Sep 26;42(11):1311–7. doi: 10.1007/s13258-020-00997-7 (PMC7567733; doi:10.1007/s13258-020-00997-7)
Supplement: Supplementary file 2 — Supplementary file2 (PPTX 1871 kb) [file 13258_2020_997_MOESM2_ESM.pptx]

## Slide 1
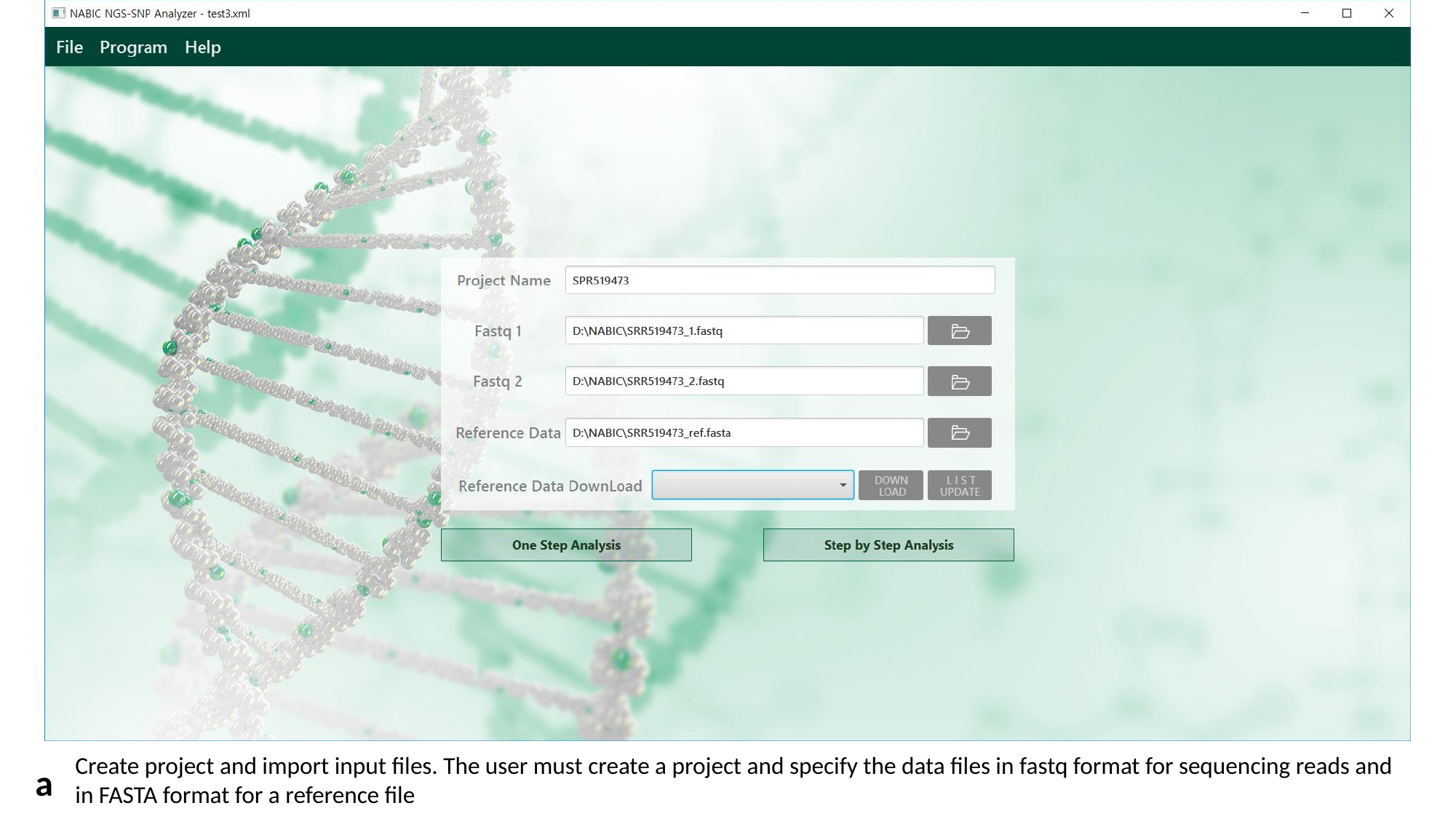

Create project and import input files. The user must create a project and specify the data files in fastq format for sequencing reads and in FASTA format for a reference file
a

## Slide 2
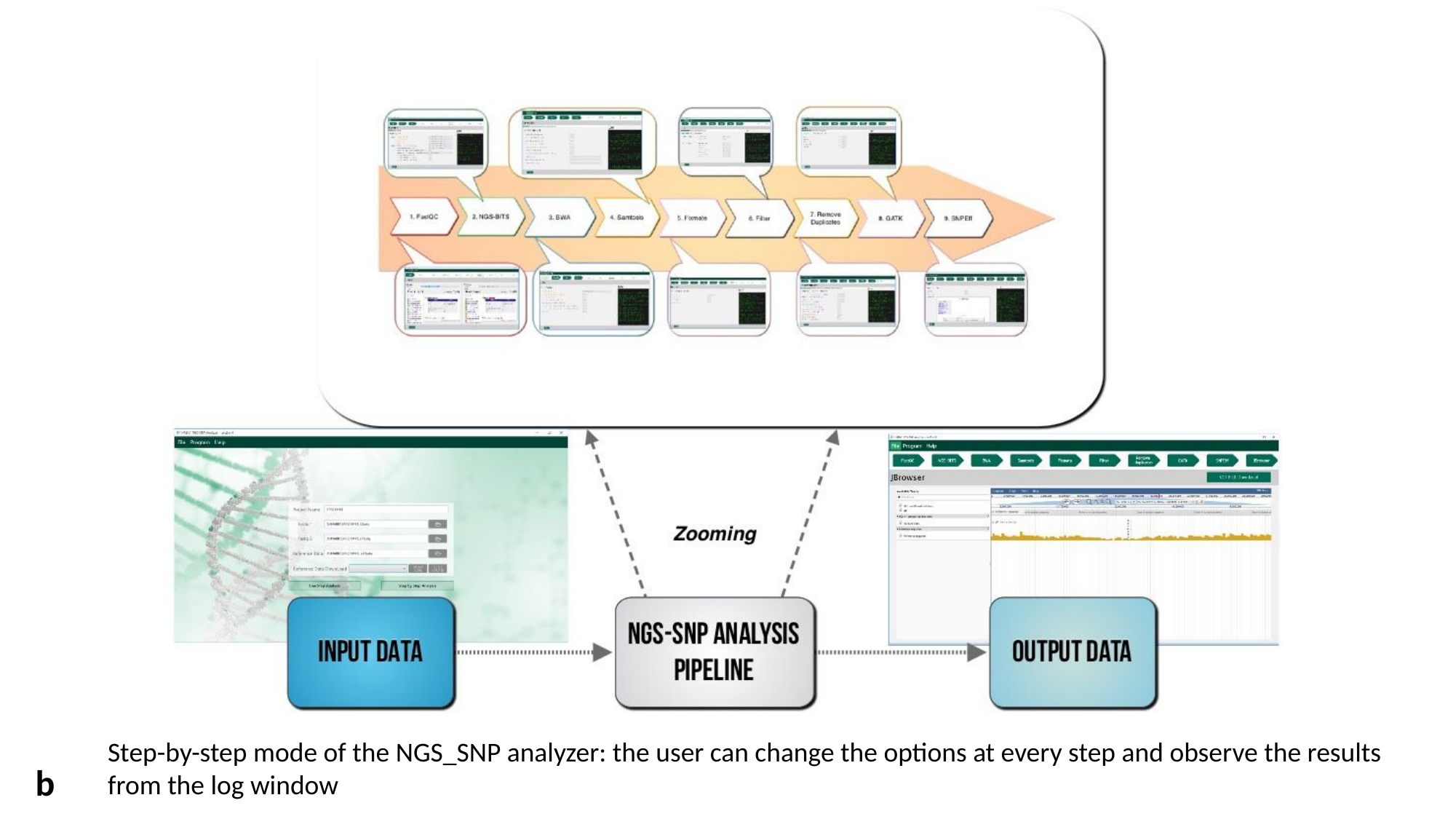

Step-by-step mode of the NGS_SNP analyzer: the user can change the options at every step and observe the results from the log window
b

## Slide 3
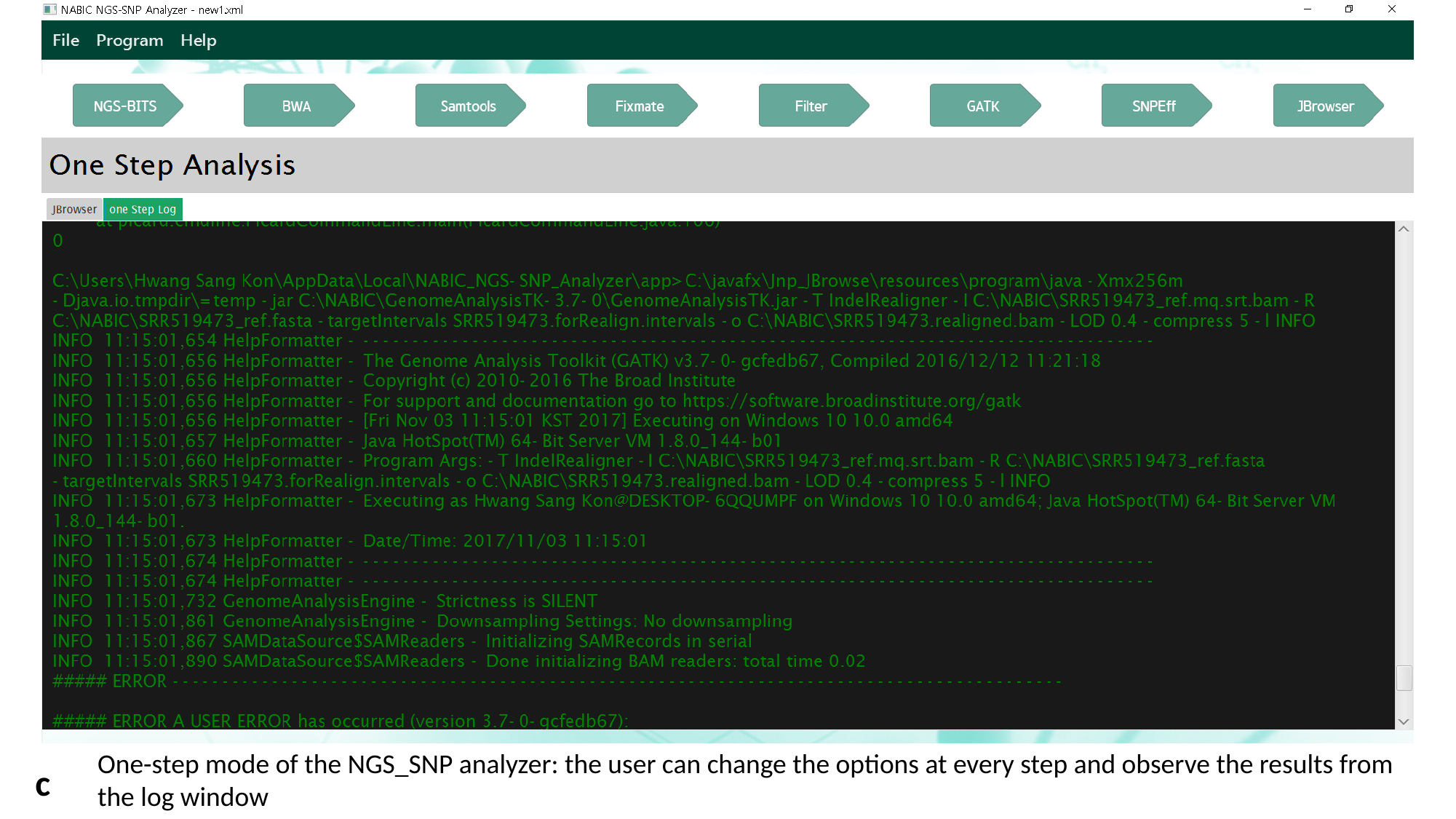

One-step mode of the NGS_SNP analyzer: the user can change the options at every step and observe the results from the log window
c
